# A Sensitive and Automatic White Matter Fiber Tracts Model for Longitudinal Analysis of Diffusion Tensor Images in Multiple Sclerosis

**DOI:** 10.1371/journal.pone.0156405

**Published:** 2016-05-25

**Authors:** Claudio Stamile, Gabriel Kocevar, François Cotton, Françoise Durand-Dubief, Salem Hannoun, Carole Frindel, Charles R. G. Guttmann, David Rousseau, Dominique Sappey-Marinier

**Affiliations:** 1 CREATIS CNRS UMR5220 & INSERM U1044, Université de Lyon, Université Claude Bernard-Lyon 1, INSA-Lyon, Villeurbanne, France; 2 Service de Radiologie, Centre Hospitalier Lyon-Sud, Hospices Civils de Lyon, Pierre-Bénite, France; 3 Service de Neurologie A, Hôpital Neurologique, Hospices Civils de Lyon, Bron, France; 4 Center for Neurological Imaging, Departments of Radiology and Neurology, Brigham and Women’s Hospital, Boston, MA, United States of America; 5 CERMEP - Imagerie du Vivant, Université de Lyon, Bron, France; University of Pécs Medical School, HUNGARY

## Abstract

Diffusion tensor imaging (DTI) is a sensitive tool for the assessment of microstructural alterations in brain white matter (WM). We propose a new processing technique to detect, local and global longitudinal changes of diffusivity metrics, in homologous regions along WM fiber-bundles. To this end, a reliable and automatic processing pipeline was developed in three steps: 1) co-registration and diffusion metrics computation, 2) tractography, bundle extraction and processing, and 3) longitudinal fiber-bundle analysis. The last step was based on an original Gaussian mixture model providing a fine analysis of fiber-bundle cross-sections, and allowing a sensitive detection of longitudinal changes along fibers. This method was tested on simulated and clinical data. High levels of F-Measure were obtained on simulated data. Experiments on cortico-spinal tract and inferior fronto-occipital fasciculi of five patients with Multiple Sclerosis (MS) included in a weekly follow-up protocol highlighted the greater sensitivity of this fiber scale approach to detect small longitudinal alterations.

## Introduction

A major challenge of neuroimaging research consists in identifying new markers that can accurately characterize pathological processes and predict clinical outcomes. Achieving this goal is particularly crucial in Multiple Sclerosis (MS), the primary cause of neurological disability in young adults and remains without well-known etiology [1]. MS is a chronic demyelinating inflammatory disease of the central nervous system, characterized by white matter (WM) lesions that are well detected by conventional MRI. However, T2 lesion load is moderately correlated with the patient clinical status leading to the development of more sensitive techniques such as diffusion tensor imaging (DTI).

DTI is a promising technique for white matter WM fiber-tracking and microstructural characterization of axonal/neuronal integrity and connectivity. By measuring water molecules motion in the three directions of space, numerous parametric maps can be reconstructed based on eigenvalues of the diffusion tensor. Among these, fractional anisotropy (FA), mean diffusivity (MD), and axial (λa) and radial (λr) diffusivities have extensively been used to investigate brain diseases [2, 3, 4, 5] such as stroke [6, 7], Parkinson disease [8, 9], brain tumors [10, 11] and also normal aging [12, 13]. In MS, DTI has proved to be sensitive enough to detect microscopic changes occurring in WM lesions, normal appearing white matter (NAWM) and subcortical grey matter (GM). Indeed, several studies have demonstrated higher MD and lower FA in lesions when compared to NAWM of MS patients [14, 15, 16] and to NAWM of healthy controls [17, 18]. In contrast, FA was increased in subcortical GM structures such as the caudate nuclei and thalami of MS patients that are supposed to reflect dendritic neurodegeneration mechanisms [19]. Overall, these findings demonstrated that WM and GM tissues are subjected to numerous microstructural alterations in MS. However, it remains unclear whether these tissue alterations result from global processes, such as inflammatory cascades and/or neurodegenerative mechanisms, or local inflammatory and/or demyelinating lesions. Furthermore, these pathological events may occur along afferent or efferent WM fiber pathways, leading to antero- or retrograde degeneration [20]. Thus, for a better understanding of MS pathological processes spatial progression, an accurate and sensitive characterization of WM fibers along their pathways is needed.

By merging the spatial information of fiber tracking [21] with the diffusion metrics derived from the tensor, WM fiber-bundles could be modeled and analyzed along their profile. Such signal analysis of WM fibers can be performed by several methods providing either semi- or automated extraction of WM fiber-bundles. Semi-automated algorithms consisted in a manual extraction of the bundle by defining a set of regions of interest (ROIs) [22, 23, 24] based on neuroanatomical knowledge. However, this task usually performed by an expert is time consuming and operator dependent. In order to overcome such limitations, fully automated algorithms have been implemented [25, 26]. These methods enable systematic, large-scale analysis of fiber bundles in large subject populations. However they remain relatively insensitive to changes affecting only a small portion of fibers within a bundle.

In this work, we introduce an automated method for the analysis of WM fascicles from DTI data, and the detection of small longitudinal changes along the fiber-tracts. Based on a Gaussian mixture model, this method provides a fine cross-sectional fiber-bundle analysis allowing the differentiation of “changed” and “unchanged” fibers of the bundle.

## Material and Methods

### Subjects

Five relapsing-remitting (RR) MS patients (4 women and 1 man, mean (±SD) age: 36.8 ± 9.5 years; media disease duration: 4.24y; max 16.5 y) (median EDSS = 2.5, range = [0–4]) and one healthy control (HC) subject (age: 24 years) were included in this study. Inclusion criteria specified that studied patients were diagnosed as RR MS and present at least one new Gadolinium-enhancing lesion during the six months preceding study enrollment. All patients were not treated with disease modifying drugs for at least one year before inclusion in the study, and remained untreated during the study period. In order to limit the nephrogenic damage risks associated to Gadolinium injection, creatinine clearance was checked every 2 weeks after inclusion. A clearance higher than 60ml/min was an exclusion criterion. This study was approved by the local ethics committee (CPP Lyon Sud-Est IV) and the French national agency for medicines and health products safety (ANSM). Written informed consents were obtained from all patients and control subjects prior to study initiation.

### MRI protocol

All subjects underwent a weekly examination for a period of two months (8 time-points from W1 to W8). MRI protocol included a DTI and a FLAIR acquisition, that were performed on a 3T Philips Achieva system (Philips Healthcare, Best, The Netherlands) with a 16-channels head-coil. The DTI image set consisted in the acquisition of 60 contiguous 2mm-thick slices parallel to the bi-commissural plane (AC-PC), and were acquired using a 2D Echo-Planar Imaging (EPI) sequence (TE/TR = 60/8210 ms, FOV = 224x224x120 mm) with 32 gradient directions (b = 1000 s.mm-2). The nominal voxel size at acquisition (2x2x2 mm) was interpolated to 0.875x0.875x2 mm after reconstruction. The FLAIR Vista 3D sequence (TE/TR/TI = 356/8000/2400 ms, FOV = 180x250x250 mm) consisted in the acquisition of 576 slices of 0.43 mm thickness oriented in the AC-PC axis with a nominal voxel size of 0.6x0.43x0.43 mm.

#### Longitudinal variations simulation

Two time-points of the control subject (W1 and W2) were used to simulate longitudinal variations. 120 different lesions were simulated on the control subject’s FA maps obtained at W2. All the lesions were generated in 6 different fiber-bundles, namely, left and right, Cortico-spinal tract, inferior-fronto occipital fasciculi and forceps major and minor of corpus callosum extracted from the atlas [27]. Small spherical variations (radius of 2 voxels) of FA values were generated according to the following equation: FA(x) = α*FA(x) where α (called reduction coefficient) varies from 0 to 1, and x is a voxel belonging to the spherical region.

### Longitudinal fiber-bundle analysis methods

**Fig 1:**
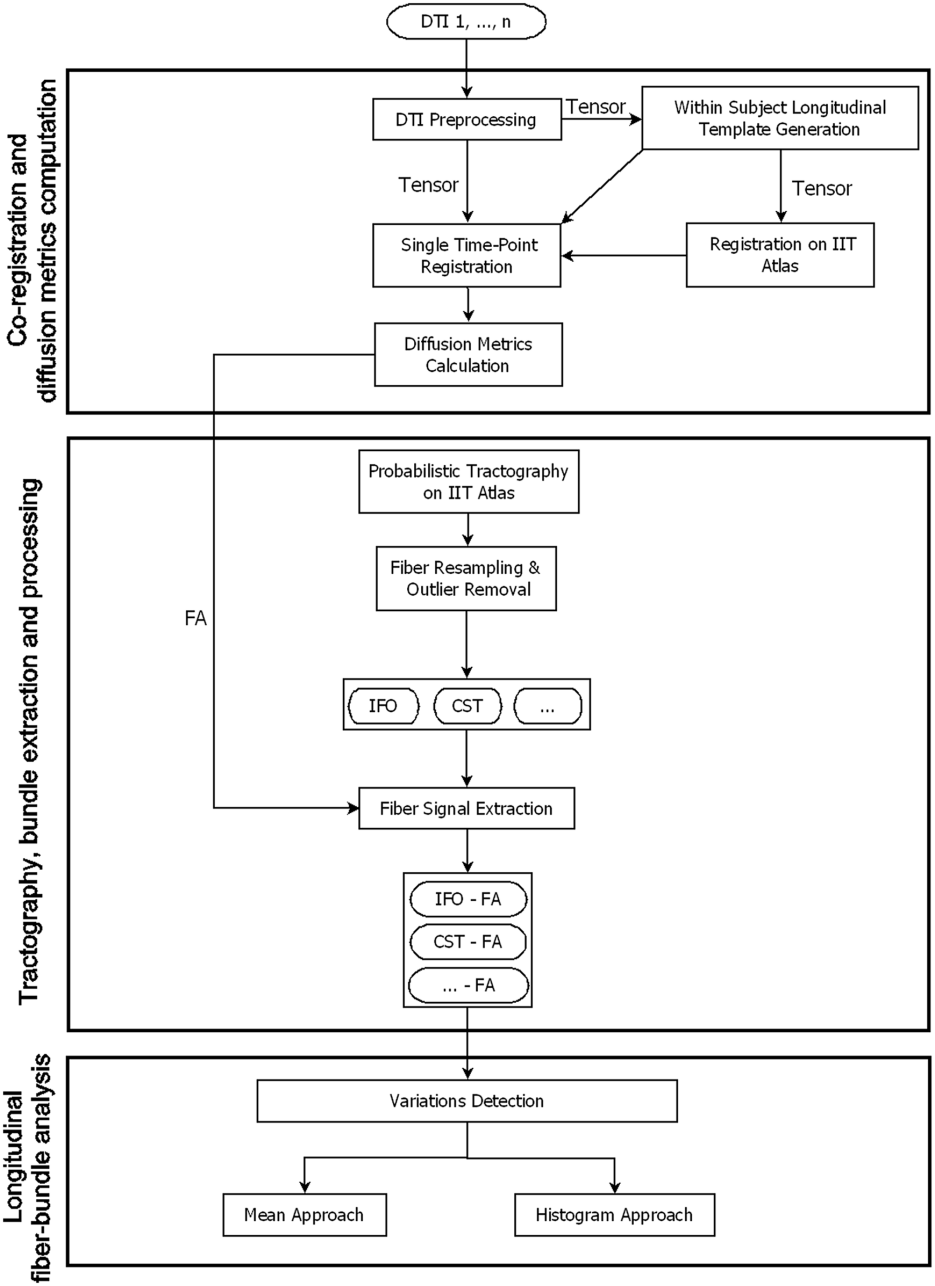
General overview of the processing pipeline for fiber-bundles longitudinal analysis. 1) Co-registration and diffusion metrics computation: DTI data were longitudinally co-registered and diffusion metrics were computed, 2) Tractography, bundle extraction and processing, 3) Longitudinal fiber-bundle analysis using both “mean” and “histogram” methods.

The processing pipeline of DTI data is composed of three steps: 1) co-registration and diffusion metrics computation, 2) tractography bundle extraction and processing, and 3) longitudinal fiber-bundle analysis (Fig 1). In the following, we assume that each subject underwent a longitudinal DTI examination. Each longitudinal acquisition is composed of *k* time-points from 1 (W_1_) to *k* (W_k_).

#### Co-registration and diffusion metrics computation

Diffusion images were processed using the FMRIB software Library (FSL) [28]. Eddy current correction was first applied on the diffusion volumes using the b0 volume (b = 0 s.mm-2) as reference. The tensor model was then fitted using the FDT module of FSL

Longitudinal data co-registration was performed using the method described in (Keihaninejad et al., 2013) based on DTI ToolKit (DTI-TK) including the following procedures: 1) generation of a patient specific template obtained from longitudinal diffusion tensor images, 2) co-registration of the resulting template to the Illinois Institute of Technology (IIT) atlas (Varentsova et al., 2014), and 3) co-registration of each time-point data into the IIT atlas space by applying the previously obtained transformations to the initial longitudinal data. The resulting images were then used to compute diffusion metrics maps (FA, MD, …).

#### Tractography, bundle extraction and processing

Probabilistic streamline tractography was performed using MRTrix [29] based on the fiber orientation density (FOD) information of the IIT Atlas. Twenty fiber bundles were extracted using 20 ROIs of the JHU atlas [27] as seed and mask.

Before the extraction of diffusion metrics, each fiber-bundle underwent a three-steps processing pipeline. The first step consisted in defining the start and end points of each fiber within the bundle. A classical K-Means algorithm [30] was applied to the extracted raw fiber-bundle to generate two different clusters, R_1_ for the starting points and R_2_ for the ending points. Fiber points were reordered from R_1_ to R_2_ and fibers that did not link the two clusters were automatically removed. In a second step we resampled each fiber with *c* = 100 equidistant points (also called nodes). The third and last step consisted in the removal of fibers that were too distant from the center of the fiber-bundle. The mean fiber of the entire bundle was first computed using the method described by Klein and coworkers [31]. Let F be a fiber-bundle F = {f_1_, f_2_, ⋯, f_m_} composed of *m* fibers of 100 points each, such as fi = {p_1_, ⋯, p_100_} 1≤i ≤ c, p_q_ = (x_q_,y_q_,z_q_) and let *M*_*F*_ = {(*p*_*μ*,1_, *p*_*σ*,1_),…,(*p*_*μ*,*c*_, *p*_*σ*,*c*_)} be the mean skeleton of the fiber-bundle where *p*_*μ*_,_*i*_ and *p*_*σ*_,_*i*_ 1≤i ≤ c represent respectively the mean and the standard deviation (SD) of computed from every point belong to the *i-th* cross-section. A fiber f_ot_ was considered an “outlier” if it contains at least one point p_r_ that was more than 3 SD away from the corresponding node in the mean fiber.

The last step consisted in the automatic extraction of the diffusion metrics from the fiber-bundle. Based on the resampled fibers, each fiber point (x_i_,y_i_,z_i_) was associated with the diffusion metric value of its corresponding voxel. Thus, every point of the fiber-bundle was associated with a set of diffusion metrics values allowing the characterization of the diffusion properties of the entire bundle. In this report, we focused on the FA metric, but the method is designed for any diffusion metrics that can be derived from the tensor.

#### Longitudinal fiber-bundle analysis

**Fig 2:**
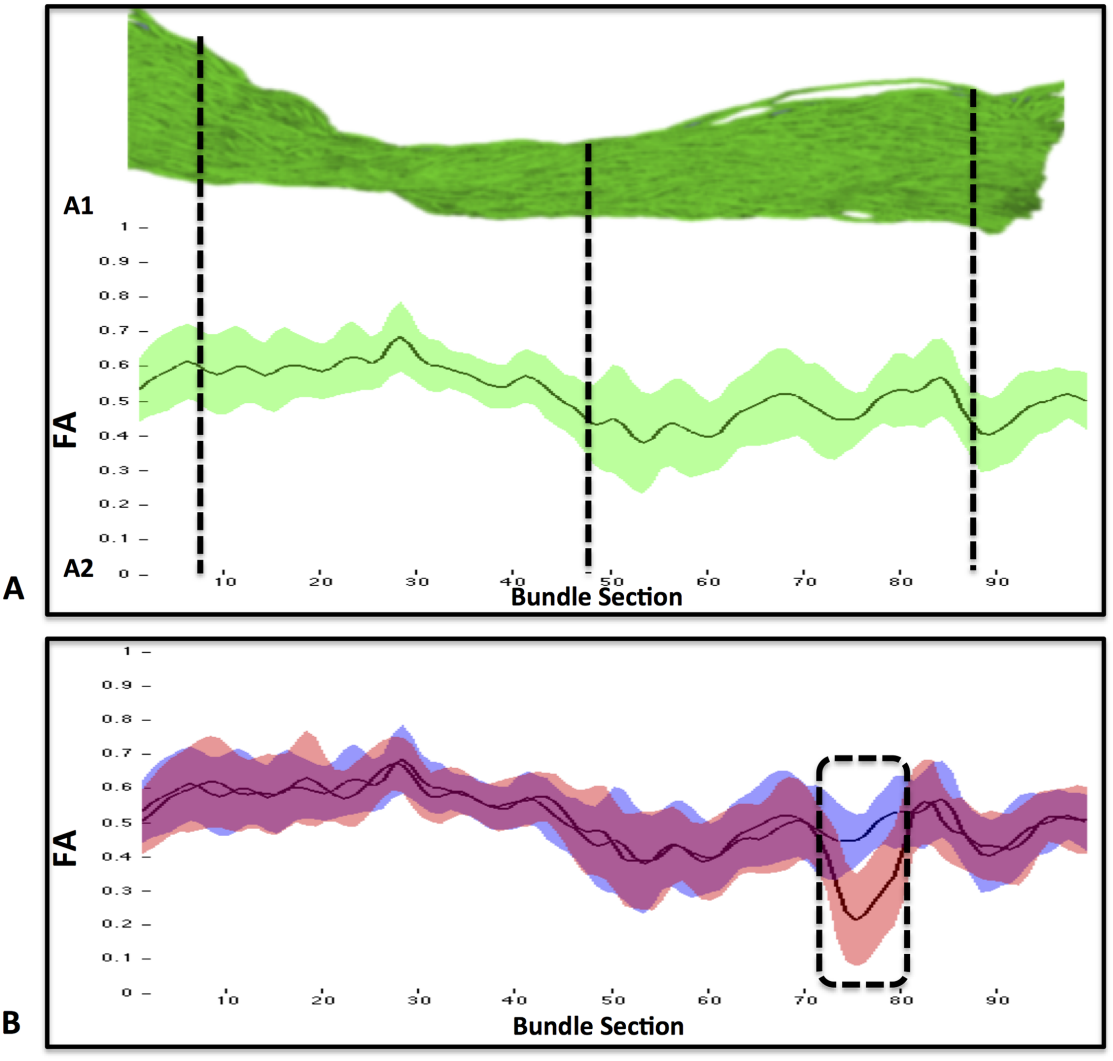
**(A1)** “Mean” cross-sectional analysis of the inferior fronto-occipital fasciculi (IFOF). **(A2)** FA values are represented by the mean (black solid line) and standard deviation (green bands) in each cross-section of the fiber-bundle. B) Longitudinal analysis of FA values between the first (blue) and fourth time-point (red) showing a significant FA decrease (no intersection in standard deviation) in several cross-sections (dashed box) of the IFOF.

Previous fiber-tract profile approaches [23, 25, 22, 32] were based on representing diffusion metrics along a given fiber-bundle by averaging the signal value at every cross-section of the bundle. This “mean” approach allowed first, to represent the mean and SD of any diffusion metric and second, to detect any changes along the fiber-bundle, as illustrated in Fig 2.

**Fig 3:**
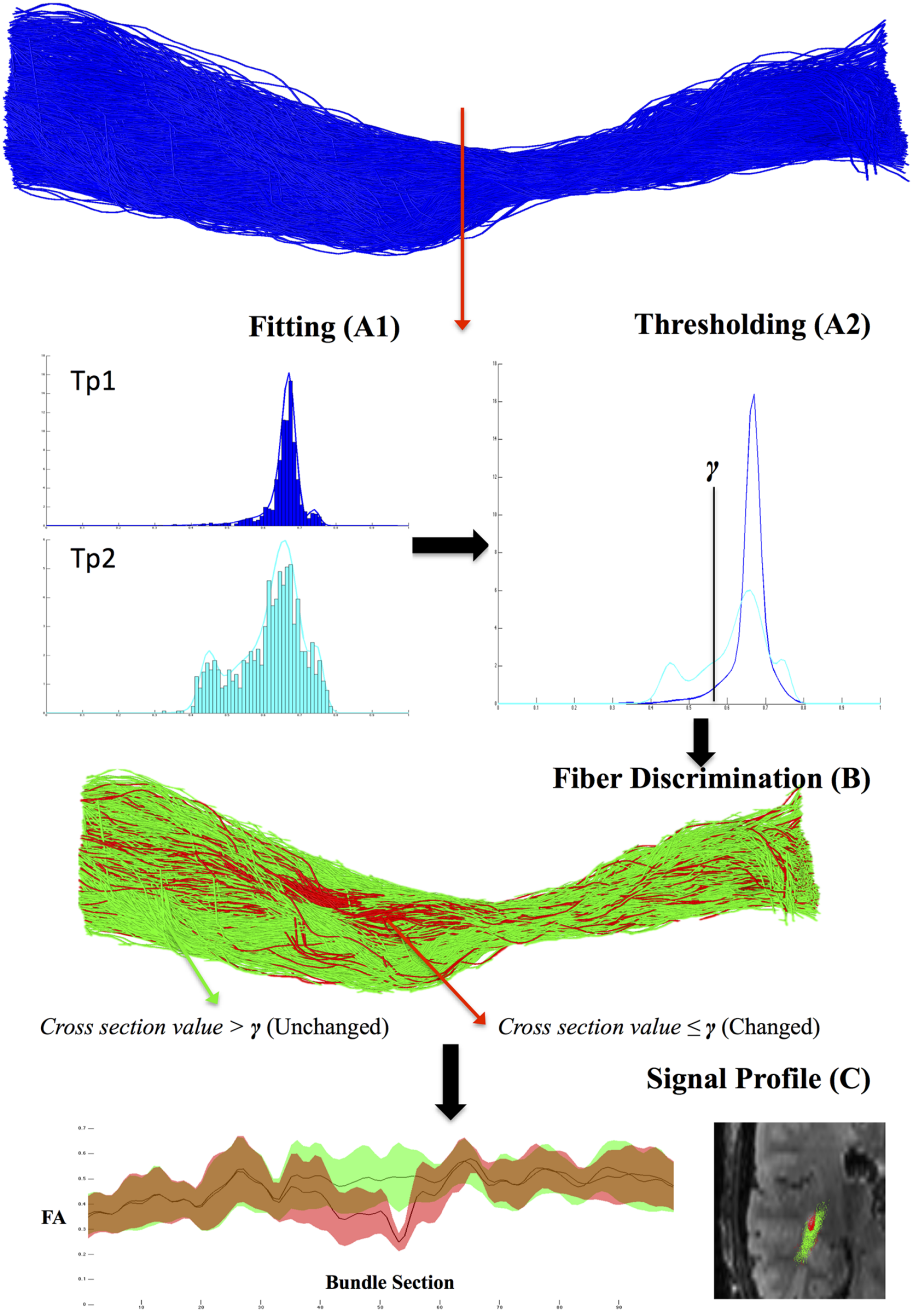
Global overview of the “histogram” approach. As first step **(A1)** the histogram of the data extracted from time point *i* and time-point *i+p* in the same cross-section are fitted using Gaussian mixture model. As second step **(A2)** our method detects a pathological longitudinal variation between the two time-points in the histogram. The obtained threshold value γ is then used to differentiate between “changed” and “unchanged” fibers **(B)**. Plotted FA signal profile of the two subset of fiber and cross-sectional view of the labeled fibers **(C)**.

In order to improve the sensitivity of the “mean” approach to detect small pathological changes along a fiber-bundle, the following “histogram” approach was developed. The histogram of FA values of a given bundle was fitted with a distribution model for every cross-section of the bundle. For a reference time-point i (W_i_) and for a successive time-point i+s (W_i+s_), two histograms were independently fitted by means of a Gaussian mixtures model (D_i_, D_i+q_) (Fig 3A1). The number of mixture components used to fit the histogram with the Gaussian model was computed by solving the following multi-objective optimization problem:

$$\begin{array}{c} \begin{array}{c} maximizen\left\{ \begin{array}{l} \text{C}(D_{i}(n))=\sum_{t=1}^{n}\text{log}(D_{i}(x_{s}))\\ \text{C}(D_{i+s}(n))=\sum_{t=1}^{n}\text{log}(D_{i+s}(x_{t})) \end{array}\right.\\ subjectton\geq \text{1, }nINT \end{array} \end{array}$$

where Di and Di+s represent the Gaussian mixture model with n component and $\sum_{\text{s}=1}^{\text{n}}\text{log}\left(\text{D}\left({\text{x}}_{\text{s}}\right)\right)$ represents the log-likelihood of the distribution model calculated using the histogram of the cross-section. Parameters of Di and Di+s were estimated by the maximum likelihood estimation (MLE) algorithm [33]. The NSGA-II algorithm [34] was used to solve the optimization problem. From the multiple Pareto’s solutions, the one with the smallest number of mixture was selected. Then, both distributions (Di, Di+s) were compared and local changes were detected by solving the following optimization problem:

$$\begin{array}{c} \begin{array}{c} maximize\\ \beta ,\gamma \end{array}P_{D}=\int_{\beta }^{\gamma }D_{i+s}\;\\ \begin{array}{c} subject\;to\;P_{F}=\int_{\beta }^{\gamma }D_{i}\leq \zeta \;\\ \;\;\;P_{D}>P_{F} \end{array} \end{array}$$ (1)

where ζ is an input value called the tolerated error of the test. If no longitudinal variations were detected between Wi and Wi+s, the problem did not admit solutions. The obtained values β and γ were then used to differentiate fibers in two subsets. In the specific case of FA (but any other diffusivity metric can be used), fibers showing β ≤ FA ≤ γ were labeled as “changed” while the other fibers were labeled as “unchanged” (Fig 3A2 and 3B). The graphic representation of the diffusion metric values along the fiber-bundle was performed as previously described using the “mean” approach (Fig 3C) [22]. For each subset of fibers (“changed” and “unchaged”), the mean and SD values of FA were computed for every cross-section of the bundle.

## Results

### Validation on simulated longitudinal variations

The “histogram” method was applied on simulated longitudinal variations of FA (described in section 2.2.1) between two time-points (W1-W2) and was evaluated by measuring true positive (TP), true negative (TN), false positive (FP) and false negative (FN) in order to compute sensitivity, precision and F-Measure. Since our Gaussian mixture model depends on two parameters (ζ and β) the method was tested with different values of ζ, {0.02, 0.05, 0.08, 0.10, 0.12, 0.14} and β was fixed to 0 in order to detect decreased FA changes as usually observed in pathological WM. As shown in Table 1, our method is strongly dependent on the parameter ζ. Low ζ values (ζ = 0.02) makes the method more conservative and a large number of FN are thus detected. In contrast, high ζ value (ζ = 0.14) results in a large number of FP. The best performance, in terms of average F-Measure (65.58%) for all the α, was reached for ζ = 0.12.

**Table 1:** Evaluation of detection performances, as measured by the sensitivity, precision and F-Measure, on simulated longitudinal lesions in function of the reduction coefficient α using different ζ values.

	*α*	0.1	0.2	0.3	0.4	0.5	0.6	0.7	0.8	0.9
**Sensitivity (%)**	ζ = 0.02	10.83	10.83	11.67	19.17	34.17	48.33	59.17	68.33	79.17
	ζ = 0.05	15.00	15.83	25.83	36.67	52.50	63.33	72.50	78.33	85.83
	ζ = 0.08	24.17	28.33	39.17	52.50	65.00	75.00	81.67	85.00	92.50
	ζ = 0.10	33.33	36.67	45.83	57.50	69.17	79.17	84.17	87.50	93.33
	ζ = 0.12	45.83	48.33	55.83	65,83	77.50	82.50	85.83	89.17	95.00
	ζ = 0.14	55.00	56.67	63.33	71.67	81.67	49.28	88.33	92.50	96.67
**Precision (%)**	ζ = 0.02	50.00	50.00	51.85	63.89	75.93	81.69	84.52	86.32	87.96
	ζ = 0.05	50.00	51.35	63.27	70.97	77.78	80.85	82.86	83.93	85.12
	ζ = 0.08	51.79	55.74	63.51	70.00	74.29	76.92	78.40	79.07	80.43
	ζ = 0.10	51.28	53.66	59.14	64.49	68.60	71.43	72.66	73.43	74.67
	ζ = 0.12	50.93	52.25	55.83	59.85	63.70	65.13	66.03	66.88	68.26
	ζ = 0.14	50.77	51.52	54.29	57.33	60.49	61.68	62.35	63.43	64.44
**F-Measure (%)**	ζ = 0.02	17.81	17.81	19.05	29.49	47.13	60.73	69.61	76.28	83.33
	ζ = 0.05	23.08	24.20	36.69	48.35	62.69	71.03	77.33	81.03	85.48
	ζ = 0.08	32.95	37.57	48.45	60.00	69.33	75.95	80.00	81.93	86.05
	ζ = 0.10	40.40	43.56	51.64	60.79	68.88	75.10	77.99	79.85	82.96
	ζ = 0.12	48.25	50.22	55.83	62.70	69.92	72.79	74.64	76.43	79.44
	ζ = 0.14	52.80	52.80	52.80	52.80	52.80	52.80	52.80	52.80	52.80

High performances were reached for α≥0.2 showing the ability of our method to detect small pathological longitudinal variations. While low performance were obtained with α = 0.1, probably due to FA variability. The “mean” method was also applied on these simulated variations. In order to check the presence of longitudinal changes, manual inspection of the obtained plot was performed to find cross-sections where no overlap between the confidence intervals (defined by one standard deviation) are visible in the signal profile generated from the two time-points. No differences were found between the two time-points with any value of α.

### Application in MS follow-up

**Fig 4:**
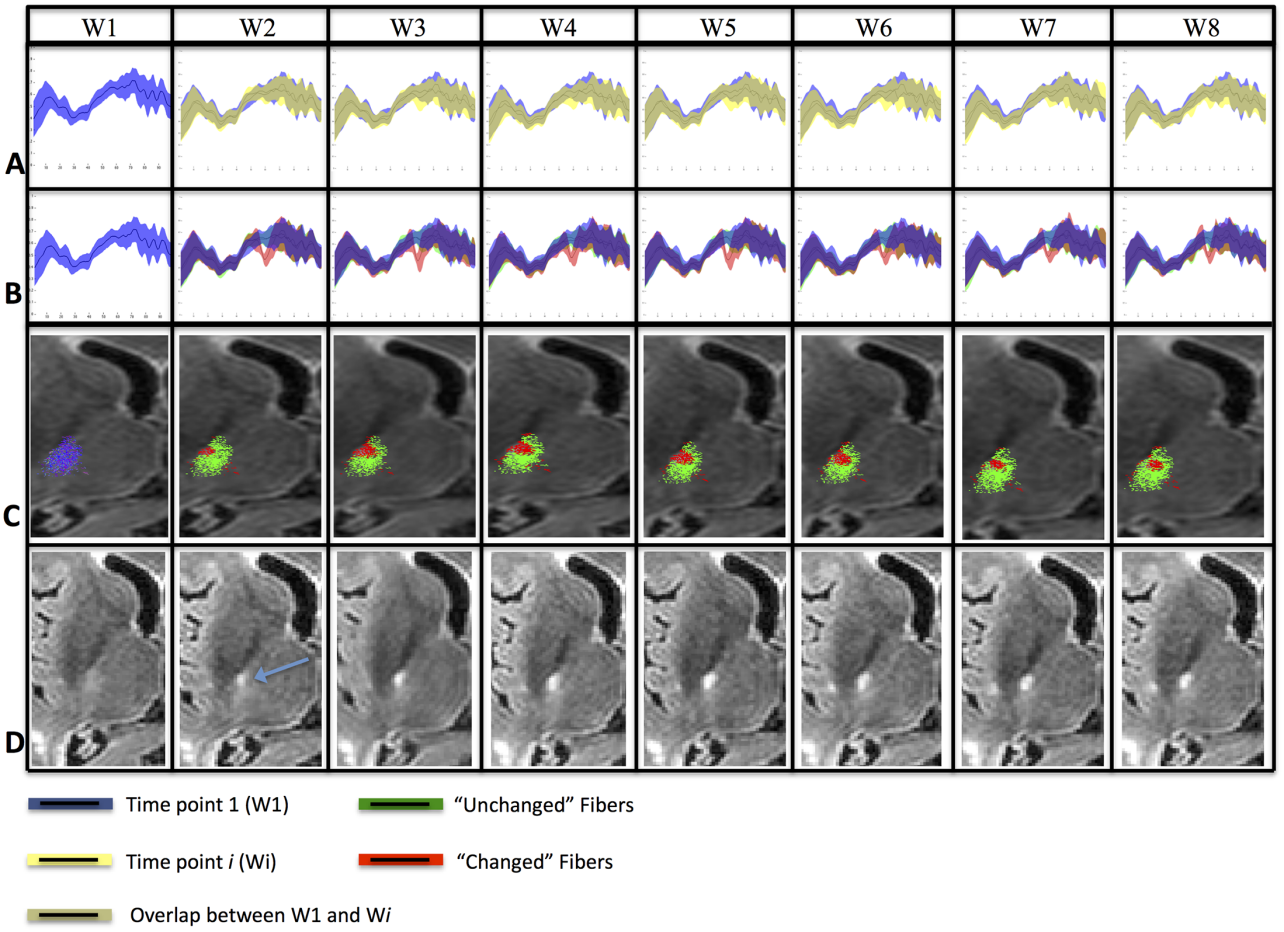
Longitudinal analyses of the FA values along the right CST of Patient1. **(A)** The “mean” method analysis showed no changes in the fiber-bundle between time-point 1 (W1, blue) and the other 7 time-points (yellow). **(B)** The “histogram” method analysis showed significant FA changes (red) between the reference time-point W1 (blue) and the others 7 time-points (W2 to W8) in different cross-sections of the fiber-bundle. **(C)** The “histogram” method allowed the distinction of “unchanged” fiber-subset (green) from “changed” fiber-subset (red) compared to the reference (W1) fiber-bundle (blue) as shown on the cross-sectional view of the CST. **(D)** FLAIR images of Patient1 showing the corresponding lesions.

DTI data of each patient were processed using our proposed pipeline including “mean” and “histogram” fiber-bundle analysis. Among the 20 fiber-bundles extracted in each patient, the Cortico-Spinal Tract (CST) and the Inferior Fronto-Occipital Fasciculi (IFOF) were selected to detect longitudinal changes. Based on our simulated results, we selected ζ = 0.12. Significant changes were validated by our neurologist (FDD) and neuroradiologist (FC) experts. An example of application of our “histogram” method on the CST of a MS patient is illustrated in Fig 4.

#### Analysis of whole fiber-bundles

**Fig 5:**
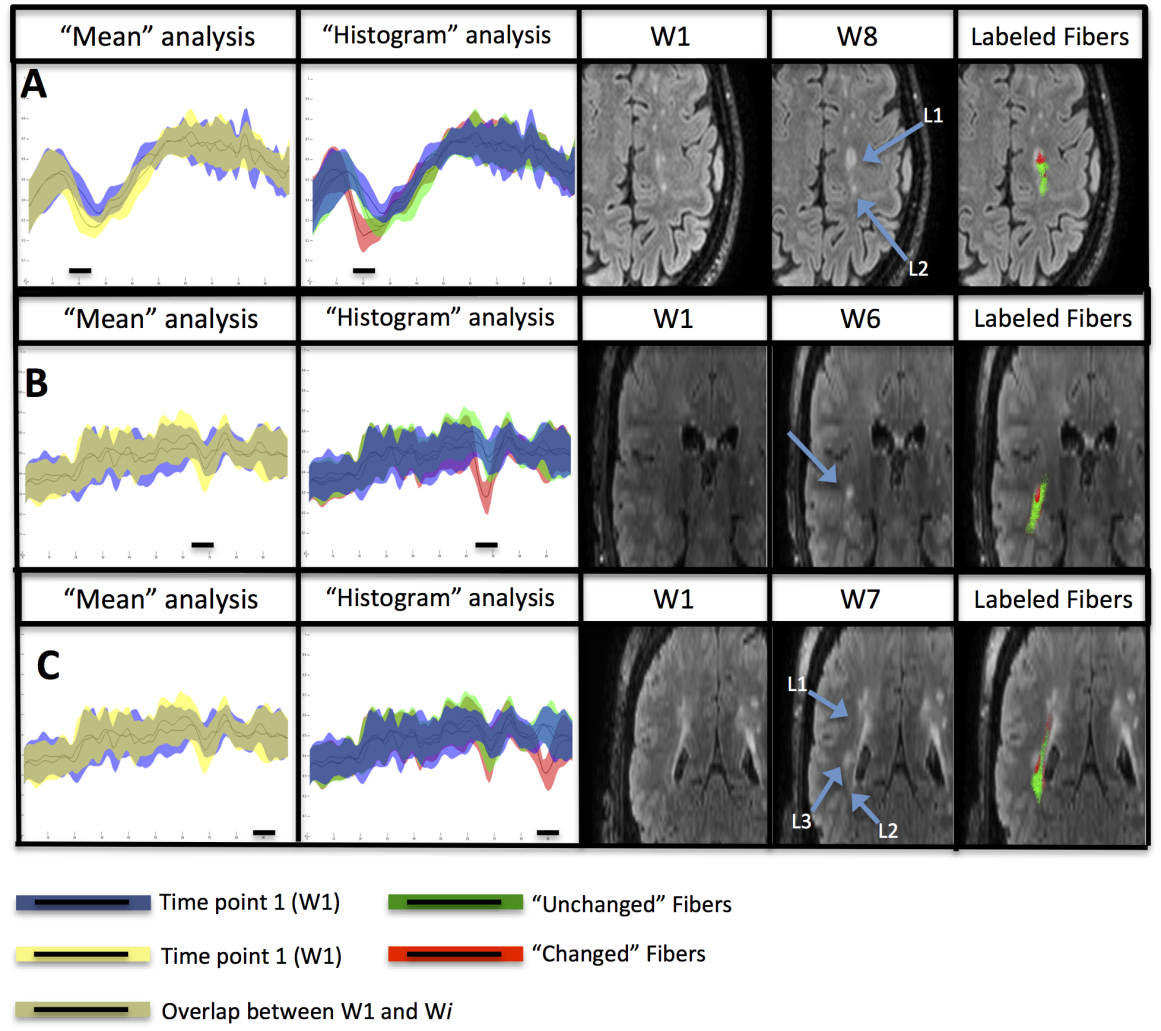
Detection of longitudinal variations by applying the “mean” and “histogram” methods. **(A)** On the left CST of Patient1 between W1 and W8 time-points, detecting a change in two preexisting lesions (L1, L2); **(B)** On the right IFOF of Patient2 between W1 and W6 detecting a new lesion; **(C)** On the right IFOF of Patient2 between W1 and W7 detecting a change in two preexisting lesions (L1, L2) and the apparition of a new lesion (L3). Lesions are shown on FLAIR images. Fiber-subsets labeled as “unchanged” (green) and “changed” (red) are shown on top of FLAIR images.

Three typical cases of longitudinal lesion evolution were selected for illustration. First, the case of two lesions preexisting at W1 in the left CST of Patient1 is shown in Fig 5A. The first lesion was well detected by the “histogram” method due to its size increase, while the second lesion was not detected due to its lack of change during the follow-up period. Second, a new lesion was detected at W6 in the right IFOF of Patient2 (Fig 5B). Third, the case of two lesions preexisting at W1 and a new lesion appearing at W7 in the same cross-section of the right IFOF of Patient2 is presented in Fig 5C. In these three cases, the “mean” method failed to detect any changes in contrast to our “histogram” method that allowed to differentiate “changed” fibers, characterized by significant longitudinal changes, from “unchanged” fibers.

#### “Changed” fiber-subsets analysis

**Fig 6:**
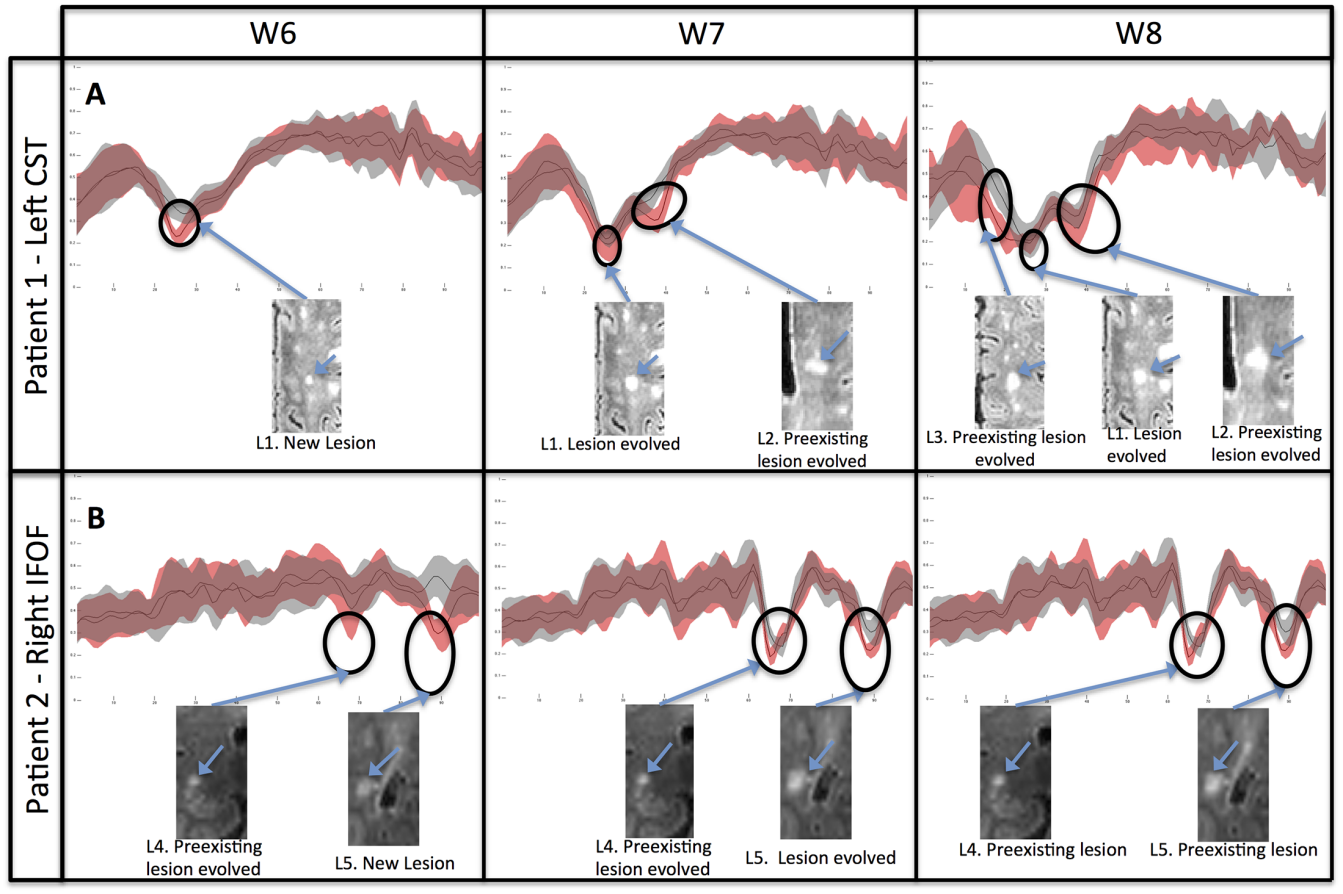
Iterative analysis of the “changed” fiber-subset of Patient1’s left CST (A) and of Patient2’s right IFOF (B) at different time-points. **(A)** Detection of a new lesion (L1) at W6 and at W8, and a preexisting lesion at W7, evolving by contaminating the CST). **(B)** Detection of a preexisting lesion (L4) and a new lesion (L5) at W6, both evolving in size and degree of FA alteration at W7, and remaining unchanged at W8.

Following this previously described identification of the “changed” fiber-subsets; we iteratively applied our method to further characterize their spatial and temporal evolutions, as illustrated in the right CST of Patient1 through the W6-W8 period (Fig 6A). The analysis of the fiber-subset’s signal revealed a new lesion occurring at W6 and evolving through W7 and W8. The “histogram” method was able to identify at W7 the progression and expansion of a preexisting lesion in a neighboring fiber-bundle of the CST, and to detect new changes at W8. Also, a second preexisting lesion was detected at W8 in another cross-section of the CST. This lesion, already present at W1, started to evolve at W8. In Fig 6B the analysis of the “changed” fiber-subset in the right IFOF of Patient2 through the W6-W8 period showed the presence of a new lesion appearing at W6 and evolved at W7 and W8. The “histogram” method was able to identify at W6 a preexisting lesion and to follow its evolution through W7 and W8 time-points.

## Discussion

The combination of fiber-tracking and DTI-derived measures, such as FA or other tensor metrics, offers a novel opportunity for the characterization of tissue properties along the WM fiber-tracks. In this work, we presented a new methodology providing automatic processing and detection of “changed” fibers subset of the bundle. The major interest of our “histogram” analysis method relies in its sensitivity to detect small FA changes at multiple locations along the fiber-bundle. In contrast to the “mean” analysis where local scale information contained in each cross-section of the bundle was lost, our method reduced the scale analysis, compatible with a “single” fiber scale. This scale reduction provided two main advantages. First, the detection sensitivity was greatly improved. Second, this fine scale approach allowed us to discriminate “changed” from “unchanged” fibers coexisting in a same bundle.

### Clinical Interest

This new method was applied in MS patients to demonstrate its clinical interest for the characterization of MS pathological processes and its potential to detect fine longitudinal tissue damages based on diffusion metrics. For example the analysis of the fiber properties along and inside the tract showed various profiles of occurrence and evolution of lesions. Indeed, WM fibers of MS patients are subjected to several and complex pathological mechanisms such as inflammation, demyelination and Wallerian degeneration that occur in various WM regions and at different time intervals. Thus, this approach could be applied to study the propagation of tissue damages along the “changed” fiber-subsets and investigate the relationship between WM lesions and their neighboring WM tracts, as well as distant cortical and subcortical GM structures. Indeed, WM lesions might have a role in deep GM atrophy as previously shown in MS patients [35, 19]. Finally, the detection of “changed” fiber-subsets based on their diffusion properties may constitute a promising tool to measure the gradient of alterations inside and along the fiber, and potentially to better understand the disease progression [36].

### Methodological limitations

Our method provided a complete, operator independent and automated processing pipeline applicable in large cohort studies. The reliability of the proposed approach stands on the accuracy and robustness of the pre- and post-processing procedures of the fiber tracking. First, each patient’s time-points images were co-registered to a common diffusion atlas in order to use the atlas tractography as a reference model for the longitudinal acquisition of the subject.

A major limitation stemming from the longitudinal registration pipeline used in this work could be the introduction of new biases. Specifically, regions affected by pathology might potentially be improperly registered due to the use of nonlinear registration.

Second, our approach provided an automatic fiber-bundle extraction by using an atlas-based extraction method. Third, the resulting bundles were post-processed to remove improper fibers generated by the tractography algorithm. This step was performed in order to improve the reliability of the local cross-sectional fiber analysis. Despite the great interest of our method to provide a better differentiation of “changed” fiber-subsets, we should keep in mind that absolute measurement of fibers number is out of reach due to intrinsic limitations of tractography algorithms [37]. However, if the tracking algorithm is compatible with crossing fibers, FA and other tensor-derived measures are not. This means that, in regions containing crossing fibers, if a difference appears in the metric, it will be attributed to all fiber-bundles traversing the region.

The capability of our method to detect small longitudinal alterations in FA maps was tested using simulated lesions generated on a control subject’s data. This test showed that our “histogram” approach is more sensitive to detect very small spherical alterations than the “mean” approach that failed to detect any of them. These results suggest that our method enables the detection of small alterations that remained undetectable by the classical “mean” approach. The sensitivity of our proposed method appeared adequate in studying MS lesions, but will require more formal sensitivity assessment in future work, to quantify its limits and potentials in this regard.

It should be noted that the proposed approach constitutes a detection mechanism; it does not perform genuine statistical analysis of the data. Indeed, the detection of change presented in this work could also been accomplished in simpler fashion: voxel-wise analysis of change could easily be combined with tractographic identification of fibers and bundles.

We expect that the strength of our approach will come to bear in situations where properties (e.g. FA) need to be grouped for voxels along a specific fiber bundle or across its cross-section. One can for instance envision increasing the sensitivity of change detection within a given fiber tract by integrating change specifically along a given tract. This is straightforward to accomplish by simple adaptation of the proposed formalism.

Since a number of recent reports in the literature have demonstrated that normal appearing white matter (i.e. tissue outside of overt lesions) plays an important role in MS [38, 39], in the future we also plan to apply our method to the assessment of more subtle changes to the properties of specific tracts.

## Conclusion

We have described a new fully automated tool for analyzing longitudinal changes in WM fiber-bundles of MS patients. Compared to previous methods developed for the characterization of fiber-tract profiles, our approach provides a fine detection of local scale longitudinal variations along the fiber-bundle, such as those expected to occur during inflammatory or neurodegenerative processes in MS patients. This new approach allows the discrimination of affected fiber-subsets within a bundle, and holds the potential for more detailed and topographically specific description of disease-induced disruption of connectivity in the brain, with implications for specific functional losses associated with disease progression.
